# Etiology, effects and management of comorbidities in multiple sclerosis: recent advances

**DOI:** 10.3389/fimmu.2023.1197195

**Published:** 2023-05-30

**Authors:** Ruth Ann Marrie, John D. Fisk, Kathryn Fitzgerald, Kaarina Kowalec, Colleen Maxwell, Dalia Rotstein, Amber Salter, Helen Tremlett

**Affiliations:** ^1^ Department of Internal Medicine, Max Rady College of Medicine, Rady Faculty of Health Sciences, University of Manitoba, Winnipeg, MB, Canada; ^2^ Department of Community Health Sciences, Max Rady College of Medicine, Rady Faculty of Health Sciences, University of Manitoba, Winnipeg, MB, Canada; ^3^ Nova Scotia Health and the Departments of Psychiatry, Psychology & Neuroscience, and Medicine, Dalhousie University, Halifax, NS, Canada; ^4^ Department of Neurology, Johns Hopkins School of Medicine, Baltimore, MD, United States; ^5^ College of Pharmacy, University of Manitoba, Winnipeg, MB, Canada; ^6^ Department of Medical Epidemiology and Biostatistics, Karolinska Institute, Stockholm, Sweden; ^7^ Schools of Pharmacy and Public Health & Health Systems, University of Waterloo, Waterloo, ON, Canada; ^8^ Department of Medicine, University of Toronto, Toronto, ON, Canada; ^9^ St. Michael’s Hospital, Toronto, ON, Canada; ^10^ Department of Neurology, UT Southwestern, Dallas, TX, United States; ^11^ Department of Medicine (Neurology) and the Djavad Mowafaghian Centre for Brain Health, University of British Columbia, Vancouver, BC, Canada

**Keywords:** multiple sclerosis, comorbidity, epidemiology, outcomes, models of care

## Abstract

Comorbid conditions commonly affect people with multiple sclerosis (MS). Population-based studies indicate that people with MS have an increased incidence of ischemic heart disease, cerebrovascular disease, peripheral vascular disease, and psychiatric disorders as compared to people without MS. People with MS from underrepresented minority and immigrant groups have higher comorbidity burdens. Comorbidities exert effects throughout the disease course, from symptom onset through diagnosis to the end of life. At the individual level, comorbidity is associated with higher relapse rates, greater physical and cognitive impairments, lower health-related quality of life, and increased mortality. At the level of the health system and society, comorbidity is associated with increased health care utilization, costs and work impairment. A nascent literature suggests that MS affects outcomes from comorbidities. Comorbidity management needs to be integrated into MS care, and this would be facilitated by determining optimal models of care.

## 1 Introduction

Multiple sclerosis (MS) is an immune-mediated disorder of the central nervous system (CNS), the effects of which the effects of which may be measured at the population-level using epidemiologic measures such as incidence, prevalence and mortality, by assessing health system use and costs, or considering the effects on the individual including symptoms, impairment and disability. The effects of MS vary between individuals and within individuals over time. Efforts to predict long-term outcomes have emphasized disease-specific features and have neglected valuable information regarding biological and biographical information regarding lifetime exposures and experiences that may account for individual variability in outcomes. A growing body of evidence implicates genetic factors, race, ethnicity, social determinants of health, health behaviors, and comorbidity, as influencing MS outcomes (1–3).

Comorbidity is of interest to clinicians caring for people with MS because it is both common and potentially preventable or treatable, unlike unmodifiable factors such as genetics, that affect MS outcomes. Herein, we define comorbidity as the “total burden of illness other than the disease (MS) of interest” (4), and focus on chronic rather than transient conditions such as infection or concussion. Distinguishing comorbidities from complications which develop secondary to the underlying disease can be difficult in some situations, particularly for psychiatric disorders, but is relevant to management strategies. In 2015, following a workshop held under the auspices of the International Advisory Committee on Clinical Trials in MS, several recommendations were made including addressing gaps in knowledge regarding comorbidity in people with MS (**Table 1**) (5). Herein, we review progress regarding those recommendations, and consequent implications.

**Table 1:** Prior recommendations for comorbidity research in multiple sclerosis.

Prior Recommendations for Comorbidity Research	Progress on Prior Recommendations
Establish age-specific, sex-specific, and ideally race/ethnicity-specific incidence and prevalence estimates for priority comorbidities in the MS population.^a^	Some age and sex-specific incidence estimates reported. Race and ethnicity-specific estimates still very limited.
Evaluate the effect of priority comorbidities on MS outcomes that are relevant from the perspective of the clinician, affected individual, health system and society.^b^	Multiple studies have examined effects of depression, anxiety, hypertension, diabetes, hyperlipidemia. Few studies have examined effects of chronic lung and autoimmune diseases.
Design observational studies that have strong methodological features. Include potential confounders or effect modifiers as covariates in studies of the effect of comorbidity on MS outcomes.	Several population-based studies have been published. However, a significant proportion of studies continue to use small, selected samples. Studies often do not include potential confounders (e.g., health behaviors) or examine effect modification.
Harmonize measurement of key data elements such as sex, race/ethnicity, clinical course, comorbidity, MS diagnostic criteria, and use common outcome measures to facilitate pooling and comparison of study findings.	No significant progress for comorbidity measurement.
Follow published guidelines when reporting study findings.	Reporting quality variable.
Conduct observational studies of comorbidity in MS in all world regions.	Several world regions remain understudied, particularly Africa.
Explore the mechanisms of the effects of comorbidity on MS as means of identifying potential approaches to mitigating their impact.	Essentially no progress.
Conduct clinical trials of the effect of comorbidity treatment on MS.	No clinical trials conducted.

a- Priority comorbidities: depression, anxiety, hypertension, diabetes, hyperlipidemia.

b- Priority comorbidities: depression, anxiety, hypertension, diabetes, hyperlipidemia, chronic lung disease, autoimmune disease.

## 2 Search strategy and selection criteria

We searched PubMed for observational studies and randomized controlled trials published from January 1, 2015 to June 12, 2022 using a strategy adapted from a 2015 publication regarding comorbidity (see **Supplemental Materials** for search terms) (5). We identified studies in the reference lists of retrieved articles, and from the authors’ personal files. A previous systematic review of the incidence and prevalence of comorbidity in MS identified few studies about comorbidity in non-White populations, subgroups defined by age and sex, or outside North America and Europe (6). Therefore, we emphasized recent articles, and those focusing on previously understudied regions or populations. We selected population-based studies of incidence and prevalence where available. The role of comorbid health behaviors has been reviewed elsewhere (7, 8).

## 3 Prevalence, incidence and comparisons with other populations

A 2015 systematic review suggested that the most prevalent comorbidities among people with MS were depression (23.7%), anxiety (21.9%), hypertension (18.6%), hyperlipidemia (10.9%) and chronic lung disease (10.0%), although the reported estimates varied (6). More recent studies also exhibit variation in the estimated prevalence of comorbidities, reflecting variations in population characteristics including ethnicity, age, and sex, and use of small, select samples. Nonetheless, findings regarding which comorbidities are the most common among people with MS who live in regions underrepresented in the prior systematic review are broadly consistent with that review. For example, a cross-sectional study from Chile found that depression/anxiety affected 34.9% of 453 participants, followed by thyroid disease (15.7%), and hypertension (11.3%) (9). In Japan, the most prevalent comorbidities for employed people with MS were astigmatism (46%), gastroesophageal reflux disease and gastric ulcer (both 36%). Twenty-five percent of individuals had hyperlipidemia and 17% had major depression (10). Africa remains underrepresented.

**Figure 1:**
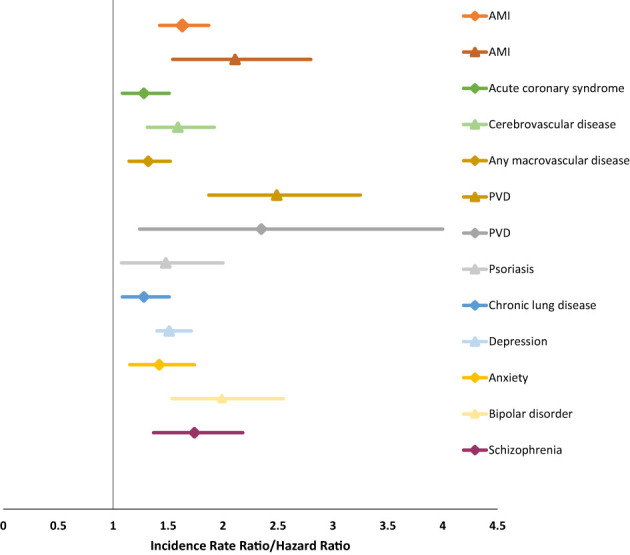
Increased incidence of comorbidity in multiple sclerosis (11–13).

Population-based studies from North America and the United Kingdom indicate that MS is associated with an increased incidence of ischemic heart disease (IHD), cerebrovascular disease, peripheral vascular disease (PVD) and venous thromboembolism post-diagnosis (**Figure 1**) (11, 12, 14). Similarly, the incidence of depression, anxiety disorders, and bipolar disorder is elevated pre- and post-MS diagnosis (**Figure 1**) (15). Younger age, female sex, lower socioeconomic status and urban residence are associated with an increased incidence of depression, anxiety disorder and bipolar disorder (13).

The incidence of most cancers did not differ between people with and without MS in recent population-based studies.^14,15^ However, bladder cancer incidence (16, 17) and mortality were elevated (17). This may reflect increased frequency of urinary tract infections in MS, use of catheters or other factors. Although people with MS reportedly experience an increased incidence of CNS neoplasms (16), mortality rates did not differ in a study involving >50,000 people with MS and >250,000 controls (17), suggesting increased ascertainment in people with MS, due to frequent imaging. Reports of an increased incidence of lung cancer and melanoma have not been consistent across studies (16–18), although a recent Mendelian randomization study suggested a causal association between MS and lung cancer (19).

Comorbidity in the pediatric MS population has received less attention. In a retrospective cohort study, 92 children with MS had a higher rate of psychiatric comorbidity (including depression, bipolar disorder, schizophrenia, anxiety, and attention deficit hyperactivity disorder) than 920 healthy children (hazard ratio [HR] 3.42; 2.46-4.76), and higher than 9108 children with non-CNS-related chronic immune-mediated diseases (HR 1.87; 1.38-2.53) (20). The burden of psychiatric comorbidity may have been underestimated in all populations because records were limited to hospital-based inpatient and outpatient care.

Other special populations with MS, including underrepresented ethnic and immigrant populations, also seem disproportionately affected by comorbidity. Although immigrants often have fewer medical conditions than the general population (‘the healthy immigrant effect’), immigrants with MS in Ontario, Canada had a higher prevalence of mood and anxiety disorders, migraines, diabetes, IHD, and chronic obstructive pulmonary disease compared to controls (21). In studies from the United States, depression and anxiety were more frequent in Black and Hispanic/Latinx individuals with MS (22, 23). In another study, hypertension and other vascular conditions were more common among Hispanic/Latinx individuals with MS, but often went undiagnosed (24). Social determinants of health such as education access and quality, health insurance, health literacy, availability of nutritious food, and the built neighborhood environment, which disproportionately affect marginalized groups may also contribute to a heavier comorbidity burden in ethnic minorities and immigrants with MS (2). Whether reductions in these disparities and early detection of comorbidities in these populations can improve outcomes has not been explored.

## 4 Etiology of comorbidity

Several general mechanisms may explain comorbidity between MS and another condition. Broadly, two conditions may co-occur due to chance, improved detection because of increased surveillance, or due to specific etiologic mechanisms. The latter may occur because of shared genetic, environmental or behavioral risk factors, because one condition and/or its treatment causes another directly, or because two conditions are caused by an unrecognized third condition.

Recent evidence suggests that MS may directly cause some comorbidities, as identified using Mendelian randomization methods. One study suggested a causal effect of MS on lung cancer (odds ratio [OR] 1.07, p=0.0082) (19) and cardiovascular diseases, including IHD (OR 1.02, p=0.03) and myocardial infarction (25, 26), but conflicting evidence for stroke (25, 26). These findings are consistent with observations that the increased risk of IHD in persons with MS is not fully explained by traditional vascular risk factors (11). By contrast, no causal effect has been identified for MS on depression (27, 28), nor for lung cancer subgroups including adenocarcinoma or squamous cell cancer (19).

Shared environmental or behavioral risk factors, such as smoking, may account for the increased co-occurrence of some comorbidities. For example, smoking is a risk factor for MS and many comorbidities, including IHD. Emotional maltreatment during childhood was associated with increased odds of having an immune-mediated inflammatory disease, including MS (OR 2.37; 1.15-4.89) and for comorbid psychiatric disorders (OR 2.24; 1.58-3.16) (29). A Mendelian randomization study recently found obesity to be a common, causal risk factor for MS and depression (27).

Shared genetic architecture may also exist for MS and comorbidities. Using large scale genome-wide association study summary data, a significant positive genetic correlation was identified between MS and amyotrophic lateral sclerosis (r_g_=0.23, p=1.1x10^-8^) (30) and inflammatory bowel disease (IBD, r_g_=0.28, p=2.01x10^-10^) (31). Future studies using Mendelian randomization methods, genetic analyses or family-based study designs may further delineate the etiology of comorbidities in MS.

## 5 Effects of comorbidities

Comorbidities may exert effects throughout the disease course from the time of symptom onset to diagnosis and to the end of life, and they may also contribute to the effects of MS on the health system and society.

### 5.1 Diagnosis

A Danish study using health administrative data found increased odds of longer delays between MS symptom onset and diagnosis in the presence of comorbidities including cerebrovascular and cardiovascular conditions, diabetes, chronic lung disease and cancer (32). The moderating role of clinical characteristics was not examined.

### 5.2 Relapses

In studies with methods including self-report, medical records review, administrative data and clinical trial datasets, the presence of multiple comorbidities has been associated with more relapses (33–35), regardless of accounting for disease-modifying therapy (DMT) use. Negative studies have had small samples or low relapse rates that reduced statistical power. Among specific comorbidities, hyperlipidemia has been associated with increased relapse rates (34, 35). An analysis of the CombiRx clinical trial found dyslipidemia associated with an increased relapse rate (HR 1.32; 1.01-1.72) (34). A 1-point increase in the Framingham Risk Score (FRS), an aggregate measure of cardiovascular risk, was associated with an 31% increased rate of relapse (HR 1.31; 1.03-1.68) (36). Associations of migraine and relapse have been inconsistent (34, 35). Evaluation of the association between psychiatric comorbidity and relapses has been more limited; in the CombiRx trial anxiety was associated with an increased relapse rate (HR 1.25; 1.01-1.55) (34).

### 5.3 Cognitive impairment

Historically, studies of comorbidity and cognition focused on depression and reported effects on processing speed and memory, the most common cognitive impairments in MS (37). In one longitudinal study, within-person elevations in depressive symptoms were associated with worse processing speed (38). Recent studies have suggested elevated anxiety symptoms are associated with poorer processing speed, working memory and new learning (39–41). Similarities and differences in the effects of anxiety and depression on cognition in MS have been reported (38, 40, 41), but both comorbid anxiety and depression should be considered given evidence for their dissociable relationships with cognition (39).

Few studies have examined other comorbidities and cognition. A cross-sectional study found dissociable relationships of anxiety and diabetes with processing speed, memory and verbal fluency tests (42). Two large retrospective cohort studies examined effects of hypertension, diabetes, hyperlipidemia and IHD, and comorbidity counts, on performance of a computer-administered test of processing speed (43, 44). The larger cross-sectional study reported those with ≥2 comorbidities had fewer correct responses but only hyperlipidemia was significantly associated with poorer scores (44). The longitudinal study reported those with ≥1 comorbidity had lower baseline test scores while those with ≥2 comorbidities showed a greater decline over time (43). However, they included depression with the vascular conditions in their comorbidity count and also identified significant effects of depression at baseline, and of incident depression during follow-up on processing speed.

Despite variable findings, recent studies suggest psychiatric and vascular comorbidities contribute importantly to cognitive impairment in MS. Prospective longitudinal studies including validated, responsive measures and assessing multiple cognitive domains are required to determine whether comorbidities contribute to specific cognitive impairments, whether shared etiological mechanisms exist for cognitive impairments in MS and comorbidities, whether comorbidities make unique contributions to cognitive impairment in MS or generalize across other conditions, and whether addressing comorbidities can reduce the risk or mitigate the severity of cognitive impairment in MS (45).

### 5.4 Physical impairment

While some small studies have not observed associations between comorbidity and physical impairments and disability (46), well-powered studies from Canada and Europe have consistently reported associations. In a study linking clinical and administrative data from 3,166 persons with MS, each additional physical (medical) comorbidity was associated with a larger annual increase in the Expanded Disability Status Scale (EDSS) score (increase: 0.18/year; 0.09-0.28) (47). Among 251 individuals with MS from Italy, a 1-point increase in the FRS was associated with an increased rate of reaching an EDSS score of 6 (HR 1.62; 1.22-3.01) (36). In a retrospective cohort of 2,725 individuals from Serbia, hypertension and diabetes were associated with a shorter time to reaching EDSS scores of 4, 6 and 7 (48). Uniquely, an Australian study examined the association between genetic polymorphisms associated with dyslipidemia and disability progression, allowing examination of the association between dyslipidemia and disability progression while limiting reverse causality (49). Individuals with ≥6 risk alleles for dyslipidemia progressed 0.38 EDSS points faster per year than those with ≤3 alleles. These studies did not account for effect of comorbidity-related therapies.

Two population-based studies indicated that psychiatric comorbidity also affected disability progression. A Canadian retrospective study that linked clinical and administrative data identified 2,312 incident cases of MS followed an average of 10.5 years (50). One-third had a mood or anxiety disorder and these individuals experienced faster disability (EDSS) progression over time (0.28/year), after covariate adjustment. A subsequent Swedish retrospective cohort study found that depression was associated with shorter time to sustained EDSS scores of 3 (HR 1.50; 1.20-1.87), 4.0 (HR 1.79; 1.40-2.29) and 6 (HR 1.89; 1.38-2.57) (51).

### 5.5 Quality of life

Comorbidity is associated with an increased incidence of fatigue, disruptive pain, and reduced health-related quality of life (HRQOL) (52). Among 949 Canadians with MS, 54.5% of those with any comorbidity had disruptive pain at baseline versus 30.7% of those without comorbidity (OR 2.70; 2.07-3.54) (53). Among specific comorbidities, fibromyalgia, rheumatoid arthritis and peripheral vascular disease were associated with the presence of disruptive pain at baseline. Over the subsequent two years, chronic lung disease, anxiety, and thyroid disease were associated with worsening pain (53). In that same study, depression (β= -0.50) had nearly the same effect on HRQOL as disability status (β= -0.52), followed closely by anxiety (β= -0.34) (54). A cross-sectional Australian study found that comorbidity accounted for 18% of the variance in HRQOL (52); psychiatric comorbidity was the strongest contributor to lower HRQOL.

### 5.6 Imaging findings

Some studies link vascular and related traits like obesity with imaging outcomes in MS, but specific results vary (**Table 2**) (55–57). For example, a higher burden of vascular comorbidities was generally associated with worse MRI outcomes in people with MS, but findings of specific comorbidities contributing to the association have varied (34, 44, 46, 55–58, 60, 64, 66). Few recent studies have examined the relationship between psychiatric comorbidities and imaging outcomes; one small study in people with MS noted lower volumes of the putamen and nucleus accumbent in people with bipolar disorder relative to without bipolar disorder (59).

**Table 2:** Summary of results of studies of comorbidity and radiological outcomes from 2015-2022.

Reference	Design Sample size	Demographic and MS characteristics	Comorbidity	Radiological outcome	Summary of Findings
Vascular and related comorbidities					
(55)	Longitudinal study 194 PwMS	Mean age: 46.7y (11.2y) 74% female: 75% RRMS	Hypertension, hyperlipidemia, CVD, diabetes, obesity/overweight	BPF, WM, GM, T2 lesion volumes, lateral ventricular volume	CVD associated with faster rates of BPF and WM loss. Hypertension was associated with higher lateral ventricle volume change Hyperlipidemia and obesity/overweight were not associated change in outcomes.
(34)	Longitudinal study 959 PwMS	Mean age: 37.9y (SD: 9.6y) 73% female 100% RRMS	hypertension, dyslipidemia, diabetes, depression, anxiety, and migraine	Combined unique lesion activity	Migraine was associated with a lower risk of combined unique lesion activity. Other comorbidities were not associated with lesion outcomes.
(56)	Longitudinal study 489 PwMS	Mean age: 71% female 59% RRMS	hypertension, CVD, smoking, obesity, type 1 diabetes	BPF, WM, GM, cortical GM, T1, and T2 lesion volumes	Hypertension and heart disease were associated with decreased total GM and cortical GM volume. Obesity was associated with increased T1 lesion volume CVD was associated with Increased lateral ventricle volume in CIS
(57)	Longitudinal study 98 PwMS	Mean age: 49.3y (12.8y) 85% female 84% RRMS	Framingham CVD risk scores, Diabetes, hypertension, hyperlipidemia, CVD, PVD, obesity	BPF	Higher Framingham risk scores were associated with greater brain volume loss over time. The association was strongest for participants with the highest brain volumes at baseline.
(58)	Longitudinal study 82 PwMS/CIS	Mean age: 32.4y (SD: 8.7y) 65% female 100% CIS/RRMS	Hypertension, hyperlipidemia, diabetes	BPF, WM, GM, cortical GM, thalamic, basal ganglia, and T2 lesion volumes	At baseline, having a vascular risk factor was associated with lower BPF, total GM, cortical GM, and WM volumes. Change in MRI outcomes was similar between PwMS with a vascular risk factor versus those without a vascular risk factor
(59)	Cross-sectional study 326 PwMS Longitudinal study 90 PwMS	Mean age: 44.2y (SD: 10.4) 65% female 90% RRMS	Diabetes, hypertension	BPF, WM, GM, cortical GM volumes	In cross-sectional analyses, diabetes was associated with lower BPF, GM, and cortical GM, and hypertension was associated with lower cortical GM. Increasing burden of vascular risk factors (diabetes, hypertension, smoking) associated with lower cortical GM. In longitudinal analysis, ≥1 vascular was associated with faster BPF loss.
(46)	Longitudinal study 51 PwMS	Mean age: 58.8y (SD: 6.4y) 55% female 100% PMS	Hypertension, dyslipidemia, obesity, diabetes, PVD or CVD	GCIPL, RNFL	Vascular comorbidity was not associated with change in GCIPL or RNFL
(60)	Cross-sectional study 573 PwMS/CIS	Mean age: 45.0y (12.4y) 71% female 100% RRMS/CIS	Migraine	BPF, T2 lesions, Gd+ lesions	Migraine was more common in PwMS or CIS than healthy people Migraine was associated with more Gd+ lesions but not volume of Gd+ lesions in PwMS or CIS
(61)	Cross-sectional study 204 PwMS	Mean age: 44.7y (11.9y) 76% female Subtype not reported	Migraine	T2 lesion volume, number of T2 lesions, Gd+ lesions, T1 hypointense lesions	Migraine was associated with fewer T1 hypointense lesions but was not associated with other MRI outcomes.
(44)	Cross-sectional study 6409 PwMS	Mean age: 47.6y (SD: 11.7y) 74% female 89% RRMS	Diabetes, Hypertension, Hyperlipidemia	BPF, GM, WM, thalamic volumes	Dyslipidemia and diabetes were associated with lower BPF Those with 2+ comorbidities had lower BPF, GM, cortical GM and deep GM volumes when compared to those with no comorbidity
(62)	Longitudinal study 469 PwMS/CIS	Mean age: 42 (SD: 10y) 70% female 100% CIS/RRMS	BMI, obesity (BMI ≥ 30)	BPF, GM	Higher BMI was associated with higher BMI was associated with faster BPF and GM atrophy
(63)	Longitudinal study 464 PwMS/CIS	Mean age: 30.8y (SD: 7.4y) 70% female 100% CIS/RRMS	BMI, obesity	Number of T2 lesions, number of Gd+ lesions, T2 lesion volume, Brain volume	No associations observed between obesity and any MRI outcomes in overall population Larger reduction in brain volume over time among obese smokers relative to normal weight smokers
(64)	Longitudinal study 768 PwMS	Mean age: 38.2y (SD: 9.4y) 73% female 100% RRMS	BMI, obesity	WM, GM, CSF	Higher BMI was associated with faster rates of GM loss, when BMI was modeled as a continuous covariate. Findings were less clear when BMI was modeled using standard cut-points.
(65)	Cross-sectional 3,046 PwMS	Mean age: 45.9y (SD: 11.7y) 73% female 70% RRMS	BMI	BPF, GM, WM, thalamic and T2 lesion volumes	No correlations between BMI and MRI outcomes
(66)	Cross-sectional 64 PwMS	Mean age: 39.8y (SD: 11.1y) 56.2% female 93.8% RRMS	BMI, obesity, TC, LDL	T2 lesion volume, Gd+ lesions	No association between BMI, TC, or LDL with lesion outcomes
(67)	Longitudinal study 522 PwMS	Mean age: 43.1y (SD: 11.9y) 72% female 70% RRMS	BMI, obesity	GCIPL, INL, ONL, pRNFL	Higher BMI was associated with faster rates of change in GCIPL. Obesity was associated with faster rates of change in GCIPL BMI/obesity were not associated with changes in the other retinal layers.
Psychiatric comorbidity					
(59)	Cross-sectional study 61 PwMS	Mean age: 40.2y (SD: 9.2y) 77% female 92% RRMS	Bipolar disorder	BPF, WM, GM, deep GM substructures (nucleus accumbens, putamen, pallidus, caudate, hippocampus, thalamus)	No differences in BPF, WM, and cortical GM volumes were observed between PwMS with bipolar disorder versus PwMS without bipolar disorder Lower putamen and nucleus accumbens observed in PwMS with bipolar disorder
Other autoimmune conditions					
(68)	Cross-sectional study 815 PwMS	Mean age: 45.7y (10.3y) 77% female 67% RRMS	Thyroid disease, asthma, type 2 diabetes, psoriasis, and rheumatoid arthritis	BPF, WM, cortical GM, MTR	Having an autoimmune comorbidity was associated with a lower BPF, cortical GM, MTR of normal appearing brain tissue and gray matter. Psoriasis, thyroid disease, and diabetes were associated with decreased BPF, cortical GM, and total GM volumes
(69)	Cross-sectional study 286 PwMS	Mean age: 42.4y (10.6y) 71% RRMS 92% RRMS	Type 1 diabetes, autoimmune thyroiditis, celiac disease	BPF, WM, GM, cortical GM volumes	Type 1 diabetes was associated with lower total GM and CM volume.

PwMS, people with MS; CIS, Clinically isolated syndrome; RRMS, relapsing-remitting MS; PMS, progressive MS; Gd+, Gadolinium enhancing; BPF, brain parenchymal fraction, GM, gray matter; WM, white matter; MTR, magnetization transfer ratio; GCIPL, ganglion cell inner plexiform layer; INL, inner nuclear layer; ONL, outer nuclear layer; pRNFL, peri-papillary retinal nerve fiber layer; RNFL, retinal nerve fiber layer. TC, total cholesterol; BMI, body mass index in kg/m^2^; LDL, low density lipoprotein; CVD, cardiovascular disease; PVD, peripheral vascular disease.

The inconsistencies across studies could reflect differences in the participant characteristics, comorbidity and imaging outcomes considered, lack of long-term follow-up in some cases, and differences in the methods for assessing comorbidity status. Most studies have been cross-sectional or small longitudinal studies (<100 patients). Larger longitudinal studies are needed.

### 5.7 Work impairment

Among 929 Australians with MS, those with any comorbidity averaged 1.2 days more lost productivity in the preceding four weeks than individuals without comorbidity (70). Similarly, a study among individuals diagnosed with MS within the last three years, found anxiety and depression were associated with both presenteeism and absenteeism (71).

### 5.8 Health care use

Comorbidity is associated with increased health care use, including hospitalizations, physician visits and prescription use. Among 2275 individuals with incident MS identified using administrative data from Saskatchewan, Canada, having any comorbidity was associated with an increased hospitalization rate (rate ratio [RR] 1.72; 1.48-1.99) (72). Nearly all comorbidities examined, including diabetes, hypertension, IHD, chronic lung disease, migraine, epilepsy and mood/anxiety disorders were associated with an increased rate of all-cause hospitalization. Another Canadian study that assessed a prevalent cohort of 4748 persons with MS and 24,154 controls tested whether mood/anxiety disorders interacted with the presence of MS to influence health care use (73). Generally, having an active rather than inactive (remitted) or no mood/anxiety disorder was associated with nearly two more days in hospital, two more physician visits and use of ≥2 prescription drug classes. The interaction between MS and mood/anxiety disorder was less than additive for physician visits, but more than additive for prescription drug use. An Italian study found that for each 1-point increase in the Charlson Comorbidity Index (CCI), the emergency room visit rates increased (HR 1.62; 1.54, 1.71) (74). However, the likelihood of an MS-related emergency room visit was not associated with the CCI score (74). All of these studies have lacked information about health behaviors, and MS clinical characteristics.

### 5.9 Mortality

People with MS continue to exhibit excess mortality versus people without MS (75); deaths due to cardiovascular and respiratory diseases, and suicide occur more often in people with MS (75). Several studies in North America and Europe observed that comorbidity is associated with an increased mortality rate among people with MS (76–79); this did not differ between immigrant and non-immigrant populations (3). Findings regarding the effects of epilepsy and chronic lung disease on mortality risk have been inconsistent, possibly due to small numbers of affected individuals in some studies (79–82). Vascular comorbidity and depression are consistently associated with increased mortality (77, 78). Two similarly designed retrospective cohort studies, one using administrative health claims data from Canada and one using primary care records and claims data from the United Kingdom, found a synergistic effect of depression and MS on mortality (77, 78), wherein the 13-14% of the mortality risk was due to the joint effect of depression and MS (77, 78). The increased prevalence of comorbidity and the increased deaths due to comorbidities in people with MS than those without MS, suggests that part of the survival gap observed could be mitigated by addressing comorbidity.

## 6 Management of MS in context of comorbidity

DMTs constitute a major aspect of MS management. Approvals for DMTs are based on explanatory rather than pragmatic randomized clinical trials (RCTs) involving carefully selected participants with MS who are closely monitored according to protocols. These select individuals do not fully represent the broader MS population treated with a DMT in the ‘real-world’ clinical setting. Specifically, persons with comorbidities are often excluded from, or underrepresented in, these RCTs. Historically, RCTs of DMTs for MS have lacked racial and ethnic diversity of participants enrolled. The disproportionate burden of comorbidity borne by non-white individuals may exacerbate this problem and add to the challenges in clinical decision-making surrounding DMT initiation for the MS population living with comorbidity.

A population-based Canadian study of 10,698 persons with MS found that a higher total comorbidity burden (≥3 vs. none) and the presence of specific comorbidities (e.g., anxiety or IHD) were associated with a 22-28% lower hazard of initiating an injectable DMT, the only DMTs widely available at that time (83). Post-DMT initiation, comorbidities may also affect adherence, persistence, and by extension, effectiveness. In Canadian studies, the presence (versus absence) of any mental health disorder with an elevated risk for earlier discontinuation of the first DMT (injectable, oral or infusion, HR 1.22; 1.03-1.44) (84). In Sweden, prior depression or antidepressant use was associated with an earlier discontinuation of interferon-beta (HR 1.51; 1.15-1.98) and fingolimod (HR 1.47; 1.04–2.08) (85).

Two Italian studies focused on the relationship between comorbidity and a DMT switch or escalation (36, 86). The larger, multi-site study, comprising 2,076 persons with MS, found that the presence (vs absence) of any comorbidity was associated with an earlier safety or tolerability-related switch from the first DMT initiated (HR 1.42; 1.1-1.9). This association differed in magnitude across the different DMTs studied (interferon-beta, glatiramer acetate, fingolimod and natalizumab; interaction test, p = 0.04) (86). The second study involved 251 MS participants at one center and observed that a 1-point increase (worsening) on the FRS was associated with a 62% higher rate of DMT escalation (HR 1.62; 1.22-3.01) (36). Combined, these studies provide evidence of temporality between prior comorbidity and subsequent DMT uptake, adherence and persistence, and likely reflect some of the real-world decision-making by persons with MS and their providers. These findings suggest that the effect of comorbidities on DMT-related MS outcomes could be substantial.

The relationship between comorbidities and the DMTs can be bidirectional (**Table 3**); by triggering onset of new comorbidities, the DMTs could augment the comorbidity burden for persons with MS. Examples include alemtuzumab, whereby autoimmune disease can occur in approximately 30% of treated persons with MS, the most common being thyroid-related (88, 89). Even among the platform DMTs considered to have better safety profiles, exposure to interferon-beta has been associated with 1.8-fold increased odds of stroke and 1.6-fold increased odds of migraine (92). While these are relative risks such that the absolute risk for the individual is likely low (especially for stroke), given how commonly these drugs are used, the population-level burden could still be considerable. Conversely, some DMTs might lower the risk of accruing new comorbidities, or perhaps improve existing comorbidities. A meta-analysis, combining one observational study and three Phase 4 open label trials found that fingolimod may improve depressive symptoms (90). Whether these improvements extend to other DMTs and reflect a direct effect of drug or an indirect effect of improvements in MS disease activity remains unknown (90).

**Table 3:** Examples of the bidirectional relationship between comorbidities and the disease-modifying therapies used to treat MS.

Direction and summary of effect	Observed effect of the comorbidity	Specific examples from the literature	Examples of key outstanding issues and/or directions for future work
Comorbidity → DMTs			
Comorbidity may affect DMT use	Delay or prevent initiation of a DMT	• ≥3 vs. no comorbidities present at MS diagnosis associated with a 25% lower risk (hazard) of starting a DMT (83) • specific comorbidities present at diagnosis - anxiety or ischemic heart disease - associated with a 22-28% lower risk (hazard) of starting a DMT (83) • only the injectable DMTs were studied (IFNB, GA)	Information is limited: • for the more recently approved DMTs (including orals and infusions) • across many world regions and healthcare settings (most cited studies are from Canada/N. Europe & universal health care settings)
	Lower DMT adherence	• Alcohol dependence associated with two-fold higher odds of poor adherence (<80% expected doses in last 30 days) to DMTs over 2-year study period (87) • only the injectable DMTs were studied (IFNB, GA)	
	Earlier DMT discontinuation	• Mental health disorders or antidepressant use associated with a 47-51% higher risk (hazard) for earlier DMT discontinuation (84)	
	Earlier DMT switch or escalation	• Presence of any comorbidity associated with a 42% higher risk of earlier safety or tolerability-related switch from first DMT used; findings differed by DMT (86) • Higher cardiovascular risk score associated with earlier escalation of DMT (36)	
DMTs → comorbidity			
DMTs may affect subsequent comorbidity burden	DMT triggers/causes onset of new disease‡	• alemtuzumab-associated autoimmune disease (affects 1 in 3 users) (88, 89) • mitoxantrone-associated cardiotoxicity and acute myeloid leukemia • several DMTs associated with severe hepatoxicity • several DMTs associated with risk of hypertension	DMT use and cancer risk largely unknown; long-term follow-up in large cohorts needed
	DMT worsens/re-activates an existing comorbidity or underlying comorbidity increases risk of another comorbidity‡	• Ofatumumab may reactivate hepatitis B virus • Macular oedema associated with several DMTs; presence of diabetes may increase risk	
	DMT improves an existing comorbidity	• Emerging work suggests that some DMTs (e.g., fingolimod) may improve underlying depression (unclear if direct or indirect effect) (90)	Limited work in this area. Inclusion of persons with comorbidities in clinical trials offers opportunity to provide further insights

DMT, disease-modifying therapy; IFNB, beta-interferon; GA, glatiramer acetate; MS, multiple sclerosis.

‡ for a complete summary of potential effects of the DMTs, see each DMTs product monograph, in addition to a recent review (91).

We need to better understand how bidirectional effects might vary by region, healthcare setting and DMT. Tackling (or harnessing) the bidirectional relationship between comorbidities and the DMTs to improve DMT-related outcomes will require a multipronged approach. This could include targeting specific populations (e.g., with anxiety) to optimize the DMT-related management of MS.

Limited information on the safety and efficacy of interventions is available to guide clinical care decisions for individuals with comorbidity. Frequently, concerns for safety and potential heterogeneity in treatment effects, result in narrow eligibility criteria for studies. Yet, multiple study designs and design features could be used to address these gaps (**Table 4**). Adaptive enrichment designs where trial enrollment criteria are adaptively learned from the trial data, could be used to address safety concerns in these populations (93). Using a group sequential approach with interim analyses, an adaptive enrichment strategy would allow for eligibility changes through the course of the trial to monitor and potentially exclude those patients based on safety concerns or those unlikely to benefit from the intervention. Rigorous Phase IV studies, when warranted, should be conducted to explicitly examine differential treatment effects. Finally, large longer-term studies are still required to establish the long-term safety outcomes (such as cancer) of the DMTs.

**Table 4:** Clinical trial designs that may address challenges and opportunities of enrolling participants with comorbidities.

Challenges	Opportunities	Designs
Safety concerns Compliance Heterogeneity of biology and treatment effects	Improve generalizability of trial results Personalized decision making Understand how effects differ due to the presence of a comorbidity or due to age Utilization of real-world evidence	Adaptive enrichment strategy design Group sequential design with interim analyses to evaluate excluding specific comorbid conditions based on safety. Phase IV studies Designing studies to evaluate safety or efficacy in individuals with comorbidity Pragmatic trials Expanding eligibility criteria Generalizable setting Master protocols to assess optimal treatment selection Registry-based randomized clinical trials Post-approval safety studies

## 7 Management of comorbidity in the context of MS

Studies of multimorbidity (presence of ≥2 chronic conditions in an individual) in the general population suggest that multiple factors influence the quality of care (94). Individuals with multimorbidity use health care more, leading to more opportunities for reassessment of their conditions and treatment modifications (95). However, the presence of unrelated or discordant conditions may lead to suboptimal care of those conditions due to competing demands or poor coordination of care across providers, and may increase treatments and financial burdens. The adverse effects of comorbidity on multiple outcomes demonstrate the importance of prevention and effective management of comorbidity. However, it can be challenging to determine if management of a particular condition (e.g., MS) needs to be modified to consider comorbidities, for instance by changing treatment targets.

Consistent with the multimorbidity literature, MS may affect diagnosis and management of comorbidities. A Canadian study relying on electronic primary care records reported that the odds of good control of hypertension or diabetes did not differ between persons with or without MS (96). However, in a population-based retrospective cohort study examining management after acute myocardial infarction, people with MS were less likely to undergo cardiac catheterization within 30 days of hospitalization (OR 0.61; 0.49-0.77), had a longer time to revascularization (HR 0.78; 0.69-0.88), and were less likely to fill a prescription for beta-blockers, high dose statins, an ACE- inhibitor/angiotensin receptor blocker, or dual anti-platelet therapy than people without MS (14). People with MS were also more likely to die 30 days after myocardial infarction (OR 1.46; 1.01-2.08). Similarly, people with MS with breast and colorectal cancer have increased mortality rates versus people without MS, even after accounting for age, sex, socioeconomic status and comorbidity (97, 98). Increased disability may account for some of this disparity but the mechanisms underlying the differences in care and outcomes are unknown.

Although comorbidity is distinct from multimorbidity, many of the same considerations for optimal care models could apply to MS care. The UK National Institute for Health and Care Excellence (NICE) guideline on multimorbidity recommends that clinicians actively consider whether an individual patient requires an approach to care that specifically accounts for multimorbidity, if the patient requests such care or if patient has certain features. For example, features such as finding it difficult to manage conditions, having mental and physical health conditions, frequently seeking unplanned care, and taking multiple medications, having frailty may warrant consideration of multimorbidity (99). **Table 5** addresses key themes emerging from the general multimorbidity literature (95) and potential considerations for persons with MS.

**Table 5:** Optimal care models and teams for managing comorbidity in multiple sclerosis (MS).

Management Concern	Optimal Approach	Specific MS Considerations
Single-disease focus adopted by healthcare systems & providers (result: fragmented, inefficient & sometimes discordant care and adverse health/quality of life consequences for person with MS and their family caregivers)	Reconfigure and technologically enhance healthcare systems – including improved balance & integration of, and communication between, specialists, generalists, and primary care providers Develop & implement healthcare policies that account for comorbidity and facilitate integration/continuity of care and person/family centered approaches Modify existing curricula to better educate new clinicians about comorbidity and its prevention & management	Include persons with MS and their family caregivers in discussions of what changes are most needed and how best to implement them Focus first on changes to healthcare systems/policies that prioritize complex comorbidity (e.g., affect multiple body systems), prevalent disease clusters (e.g., MS with depression), and/or comorbidity deemed most important to persons with MS/family caregivers Expand MS-focused interdisciplinary care teams that address identified priorities, collaborative (health & social) care needs, and support self-management, medicines management, and healthy behaviours including those relevant to comorbidity Conduct research on implementation efforts and include outcomes important to persons with MS/family caregivers (not only healthcare use, costs, barriers, and facilitators)
Single-disease focus adopted by existing clinical practice guidelines (result: failure to modify treatment & clinical/lifestyle recommendations to address disease-disease, disease-drug & drug-drug effects; increased treatment burden for person with MS and their family caregivers)	Comprehensive person/family-centered approach, considering heterogeneity of comorbidity present and care goals, values, and preferences of person with MS and their family caregivers Consider how management/treatment of a single condition might be modified to mitigate treatment burden and adverse health & quality of life outcomes associated with co-occurring chronic conditions (especially discordant comorbidity), medications and frailty (e.g., UK NICE guideline on multimorbidity, https://www.nice.org.uk/guidance/ng56)	Conduct research to illuminate MS relevant care gaps/concerns in current MS-specific clinical practice guidelines as well as guidelines for prevalent comorbidity in MS (ensure participation of interdisciplinary healthcare providers, persons with MS/family caregivers throughout research) Examine and modify (where supported by evidence) current MS clinical practice guidelines to acknowledge the complexity and burden of care imposed by comorbidity, medicines management & frailty (including input from interdisciplinary healthcare providers, persons with MS/family caregivers) Examine and recommend areas to modify (where supported by evidence) current clinical practice guidelines for prevalent comorbidity in MS (including input from interdisciplinary healthcare providers, persons with MS/family caregivers) Conduct research on implementation efforts and include outcomes important to persons with MS/family caregivers (including treatment burden, cost, non-adherence, care experience)
Socioeconomic and racial, and ethnic inequalities in availability, accessibility, affordability and continuity of care (result: even greater fragmentation of care, treatment burden & poorer health and quality of life outcomes for those who face the highest incidence of comorbidity and at younger ages)	Consider how changes to healthcare systems & policies and clinical practice guidelines specifically address (or potentially worsen) inequalities in the management of comorbidity among diverse age, sex, racial/ethnic populations Prioritize funding initiatives to address current research gaps in the optimal prevention, treatment & management of comorbidity in younger-aged and racially/ethnically diverse populations (particularly given recent trends in the incidence/prevalence of comorbidity/multimorbidity and unique challenges faced by these populations)	Ensure that above considerations and implementation research efforts include persons with MS from diverse age, sex, racial/ethnic populations and consider their unique comorbidity care needs (including comorbidity burden, barriers to optimal self-care and medicines management) Conduct research to better understand prevalence of comorbidity clusters in MS and their association with MS care and physical/cognitive/quality of life trajectories among diverse age, sex, racial/ethnic populations

Important caveats: Evidence base for the most appropriate interventions for the treatment and management of comorbidity/multimorbidity is generally weak and more robust longer-term investigations are needed (though there are many challenges to conducting this research and barriers (at patient, system, provider level) to implementing findings into practice) (95).

Screening for depressive and anxiety disorders can be accomplished using any of several valid and reliable scales (100), and even single scale items (e.g. “I felt depressed”, “I felt like I needed help for my anxiety”) can perform effective screening (101). In the general population, routine screening is recommended for hypertension, diabetes, lipid disorders and obesity (**Table 6** depicts US-based guidelines). However, optimal intervals for screening people with MS for comorbidities are unknown.

**Table 6:** US preventive services task force recommendations^a^.

Condition	Who to Screen	How to Screen	Interval for Screening
Diabetes/Pre-diabetes	Adults 35-70 years who are overweight or obese	Fasting plasma glucose OR HbA1c OR oral glucose tolerance test	Every 3 years
Hypertension	Adults ≥18 years	With office blood pressure measurement. Obtain out of office measurements to confirm diagnosis.	Every 3-5 years if not at increased risk Every year if at increased risk (overweight, obese, high normal blood pressure, Black race) or ≥40 years
Lipid Disorders	Adults ≥40 years who do not have known cardiovascular disease, have no symptoms of cardiovascular disease, and have ≥1 risk factor (diabetes, hypertension, smoking, dyslipidemia)	Blood test for cholesterol and triglycerides	Uncertain Every 5 years, shorter for those with lipid levels close to needing treatment, longer intervals if repeatedly normal levels, not at increased risk
Obesity	Adults with body mass index ≥30	Measured height and weight	

Adults without a history of cardiovascular disease use low to moderate dose statin to prevention cardiovascular disease events and mortality if: they are aged 40-75 years, have 1 or more of dyslipidemia, diabetes, hypertension, smoking, and 10-year risk of cardiovascular event of ≥10%.

Pharmacologic and psychotherapy approaches are effective for managing depressive disorders in MS but limited evidence is available regarding anxiety disorders (102). In the general population, a scoping review involving 34 articles examining the association between physical activity or exercise and vascular comorbidity found lower levels of physical activity associated with a greater burden of comorbidity, as well as the converse association (103). As well, interventional studies involving aerobic or resistance exercise reduced serum triglyceride levels, and improved blood pressure and glucose tolerance (103). Promoting physical activity may likewise improve vascular comorbidity control in MS.

Benefits of pharmacologic treatment of vascular comorbidities on MS outcomes are poorly understood, but one open-label study found treatment of metabolic syndrome with either metformin (n = 20) or pioglitazone (n= 10) was associated with a reduced number of new/enlarging T2 lesions and gadolinium-enhancing lesions, and an increase in regulatory T cells (104). Understanding of the relationships between comorbidity and MS outcomes is critical for the design of clinical trials that test the impact of effectively managing comorbidity on MS-specific outcomes. The importance of clinical trials in establishing whether this approach will be effective cannot be underestimated; a beneficial effect on outcomes should not be assumed. This is illustrated by an examination of the role of lipids in murine experimental autoimmune encephalomyelitis (EAE), in which neither increasing nor decreasing blood cholesterol levels affected peripheral immune responses or infiltration of lymphocytes into the CNS or progression of EAE (105).

## 8 Studying comorbidity interventions

Pragmatic trials are a broad class of trials implementing a range of design features, including eligibility criteria, recruitment, setting, organization, flexibility of delivery and adherence, follow-up, and primary outcome and analysis, to increase applicability to real world settings (106). Trials can be designed with varying numbers and degrees of these features incorporated. One example of incrementally increasing the pragmatism of a trial would be to expand the eligibility criteria to include individuals with comorbidities and relaxing age restrictions. Pragmatic features can be incorporated into all aspects of a trial. For example, a registry-based clinical trial, a trial that use registries as a platform for recruitment, data collection, randomization, and follow-up, allows for greater generalizability of findings, increased efficiency in the conduct of a trial and increased ability to examine potential heterogeneous treatment effects (107). Registry-based trial designs could efficiently examine the effects of comorbidity treatment on MS. While not without limitations, a critical need exists to consider these designs in MS clinical trials and generate evidence that is more representative of the broader MS population.

## 9 Conclusions

Numerous comorbidities affect persons with MS more often than individuals without MS. At the level of the individual, comorbidity is associated with greater physical and cognitive impairments, lower health-related quality of life, and increased mortality. At the societal level, comorbidity is associated with increased health care utilization, work impairment, and costs. A nascent literature suggests that MS affects the care of, and outcomes from comorbidities. Comorbidity management needs to be integrated into MS care, and this would be facilitated by determining optimal models of care.

## Author contributions

RM - conceptualization, methodology, writing - original draft, writing - review & editing. JF - writing - original draft, writing - review & editing. KF - writing - original draft, writing - review & editing. KK - writing - original draft, writing - review & editing. CM - writing - original draft, writing - review & editing. DR - writing - original draft, writing - review & editing AS - writing - original draft, writing - review & editing. HT - writing - original draft, writing - review & editing. All authors contributed to the article and approved the submitted version.
