# Supplementary material for: Etiology, effects and management of comorbidities in multiple sclerosis: recent advances
Source: Front Immunol. 2023 May 30;14:1197195. doi: 10.3389/fimmu.2023.1197195 (PMC10266935; doi:10.3389/fimmu.2023.1197195)
Supplement: Supplementary file 1 [file DataSheet_1.docx]

Supplementary Material

Etiology, Effects and Management of Comorbidities in Multiple Sclerosis: Recent Advances

Ruth Ann Marrie^*^, MD, PhD, John D Fisk, PhD, Kathryn Fitzgerald, ScD, Kaarina Kowalec, PhD, Colleen Maxwell, PhD, Dalia Rotstein, MD, MSc, Amber Salter, PhD, Helen Tremlett, PhD

*** Correspondence:** Corresponding Author: rmarrie@hsc.mb.ca

# Supplementary Methods

**This strategy was based on a prior review in which the comorbidities were selected by consensus of individuals with expertise** in clinical neurology, epidemiology and clinical trials. They included diabetes, hypertension, hyperlipidemia, ischemic heart disease, valvular disease, cardiac arrhythmias, congestive heart failure, cerebrovascular disease (stroke, transient ischemic attack), peripheral vascular disease, autoimmune disease (alopecia areata, ankylosing spondylitis, autoimmune thyroid disease, bullous pemphigoid, celiac disease, dermatomyositis, idiopathic thrombocytopenic purpura, inflammatory bowel disease, myasthenia gravis, pemphigus vulgaris pernicious anemia, polymyositis, primary adrenocortical insufficiency, primary biliary cirrhosis, psoriasis, rheumatoid arthritis, Sjogren’s syndrome, systemic lupus erythematosus systemic sclerosis, uveitis, vitiligo, Wegener’s granulomatosis), chronic lung disease (asthma, chronic obstructive pulmonary disease), gastrointestinal disease (gallbladder and biliary tract disease, gastroesophageal reflux disease, irritable bowel syndrome, liver disease, peptic ulcer disease), renal disease, visual disorders (cataracts, glaucoma, retinal disease), musculoskeletal disorders (crystal arthropathies, fibromyalgia, osteoarthritis,), epilepsy, renal disease, cancer and psychiatric comorbidity (alcohol abuse, anxiety, bipolar disorder, depression, personality disorders, psychosis, substance abuse).

**Search Strategy**

multiple sclerosis [mesh] NOT review AND (("quality of life" OR mortality OR survival OR disabil* OR progress* OR "EDSS" OR function* OR impair*) OR prognosis OR (MRI OR "magnetic resonance imaging") OR (relaps* OR reoccurr* OR recur*) OR (absenteeism OR presenteeism OR employ*) OR (hospitalization) AND (comorbid* OR hypothyroidism or myxedema or hyperthyroidism or thyrotoxicosis or "Graves disease" or thyroiditis or Hashimoto's or "rheumatoid arthritis" or "Felty syndrome" OR rheumatoid nodule OR Rheumatoid vasculitis OR adult onset Still's disease OR dermatomyositis OR polymyositis OR idiopathic inflammatory myopathies OR myositis OR ankylosing spondylitis or spondylitis ankylosing OR vertebral ankylosis OR spondyloarthropathy OR bechterew* OR Marie-Strumpell* OR inflammatory bowel disease OR IBD OR Crohn's OR ulcerative colitis OR uveitis OR uveiti* OR panuveitis OR iridocyclitis OR iridocycliti* OR "anterior scleritis" or iritis or iriti* or pars planitis or lupus or "lupus erythematosus" or "systemic lupus erythematosus" or "lupus vulgaris" or psoriasis or "palmoplantaris pustulosis" or "pustular psoriasis" or pemphigus or "pemphigus vulgaris" or "phemigus foliaceus" or "primary biliary cirrhosis" or "liver cirrhosis" or biliary or "bullous pemphigoid" or pemphigoid or myasthenia or "myasthenia gravis" or "anti-acetylcholine receptor antibody" or AChR or MuSK or "anti-muscle specific kinase antibody" or "seronegative MG" or "seronegative myasthenia gravis congenital" or myasthenia or "congenital myasthenic syndrome" or "systemic sclerosis" or scleroderma or "pernicious anemia" or "atrophic gastritis" or "type A gastritis" or "macrocytic anemia" or "cobalamin deficiency" or "vitamin B12 deficiency" or "intrinsic factor deficiency autoimmune" or "adrenal-cortex-diseases" or "adrenal-gland-diseases" or "adrenal insufficiency" or gastritis or "idiopathic thrombocytopenic purpura" or ITP or thrombocytopeni* or "Addison's disease" or "Addison disease" or "primary adrenal insufficiency" or "primary adrenocortical insufficiency" or "Wegener granulomatosis" or vasculitis or "wegener vascul*" or "small vessel vascul*" or celiac or sprue or "gluten sensitive enteropathy" or "villous atrophy" or antigliadin or endomyseal or "tissue transglutaminase" or alopecia* or vitiligo or leukoderma or leucoderma or "Sjogren's syndrome" or cancer or neoplasm or carcinoid or leukemia or lymphoma or malign* or melanoma or myeloma or hypertension or diabetes or arrhythmia or cerebrovascular or stroke or “high blood pressure” or “peripheral vascular disease” or seizure* or epilepsy or convuls* or asthma or “chronic obstructive pulmonary disease” or bronchitis or emphysema or osteoarthritis or fibromyalgia or ‘renal insufficiency’ or kidney failure or “kidney insufficiency” or “sleep apnea” or “restless leg” or cataract or glaucoma or anxiety or depression or bipolar or schizophrenia)

**
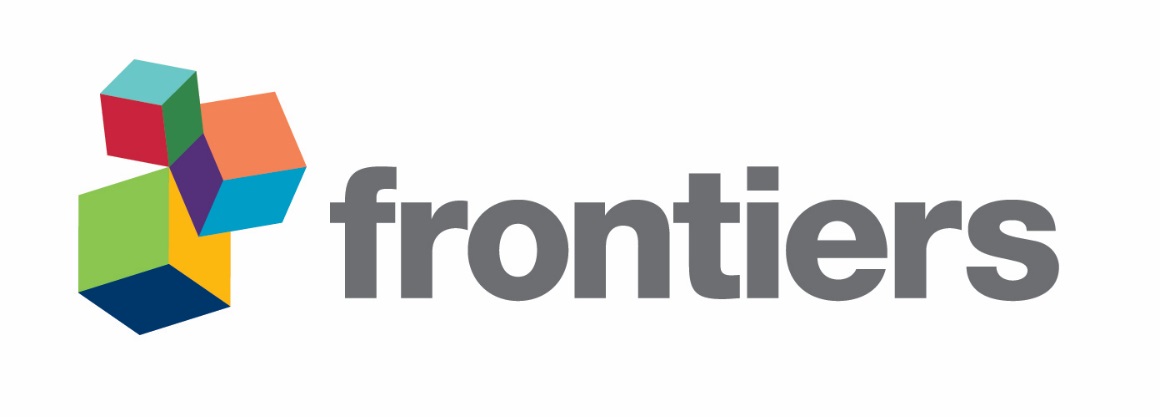
**
